# To Our Contributors

**Published:** 1874-05

**Authors:** 


### Editorial.

### TO OUR CONTRIBUTORS.

We can not but express appreciation of and gratification for the assistance our contributors have given, and are still giving us by the use ofthe pen. Comparatively few in the profession, however, seem to comprehend the responsibility that rests upon them in this matter.

In nearly all the occupations of human life, and especially in the professions, the pen is one of the most potent instrumentalities employed, or perhaps that can be employed. How important then that the ability to use it should be cultivated, and exercised too. Writing is a stimulus to systematic and accurate thought, and a means of transmitting our thoughts to others. And now our profession, in its rapidly developing stage, requires this kind of cultivation and stimulus. There are many dentists who write well, and yet very seldom make the attempt. To all such we say, remember that you owe a duty to your profession, that you are not performing. "Freely ye have received, freely give."

There are some who do occasionaly write, and still do not exercise the care they ought ; and this too in reference to systematic and close thinking, as well as to the manner of expression and construction of language. Care on the part of writers, will remedy such defects. We have occassionally had articles sent in without any caption, and sometimes with little or very imperfect punctuation.

We would suggest and urge that a great many more in the profession write, and let us all give more attention to the science and art of writing. Many will throw off an article hurriedly, and carelessly, and then ask the editor to please correct and put it up in good shape. Some would be rather astonished to see their matter printed as written. Many of the best writers of the world have subjected there manuscripts to six to ten revisions, and rewritings.

Is the dental profession of so little importance or worth, that hasty written, disjointed productions are sufficient to answer its requirements, and to establish a reputation for the members, for their attainments in science and art.

